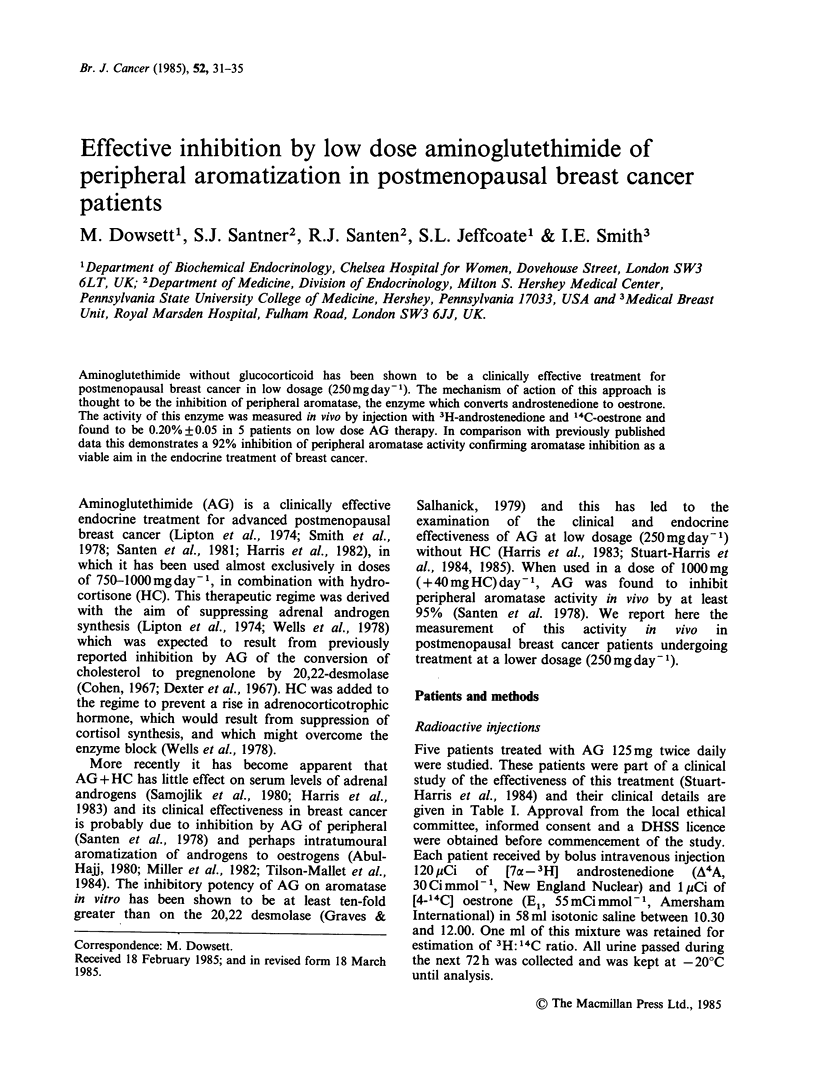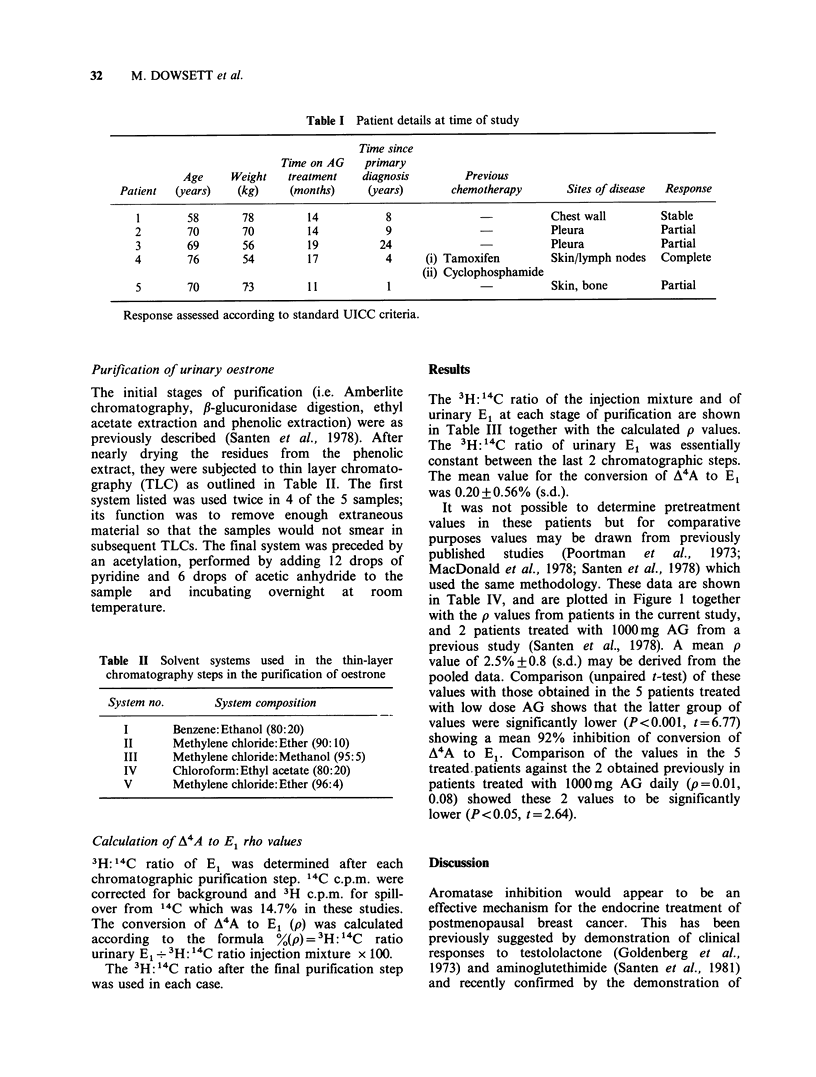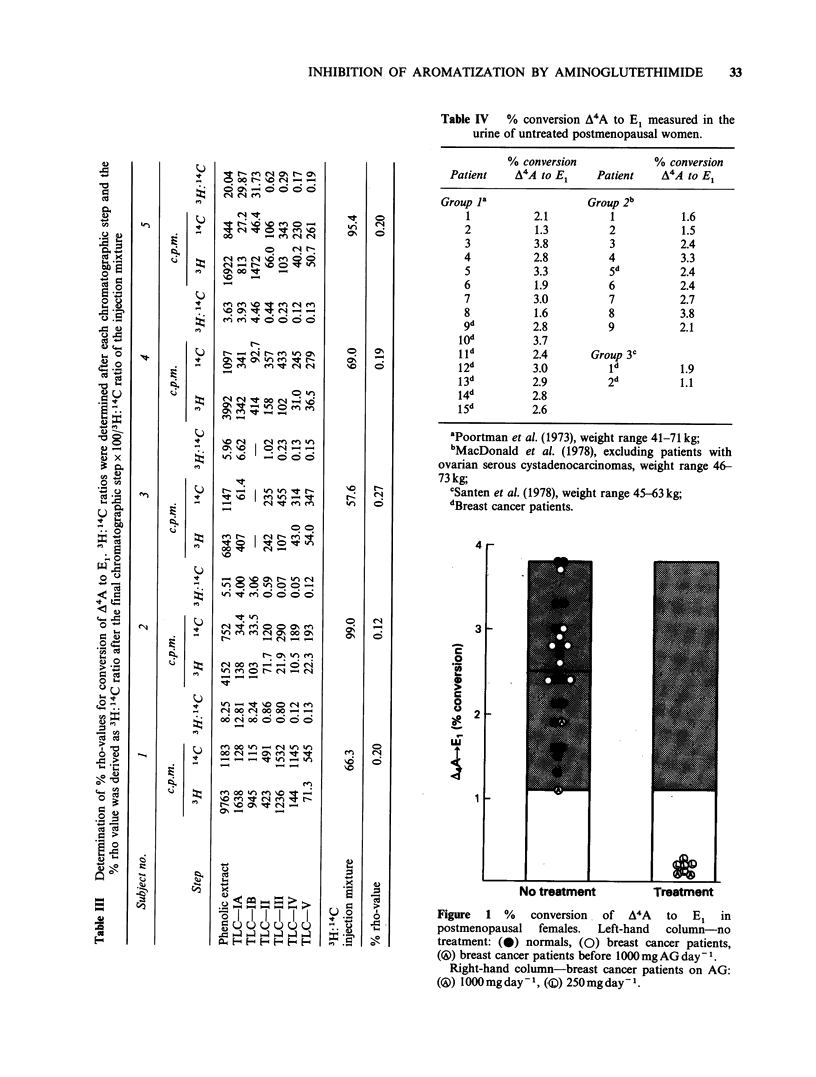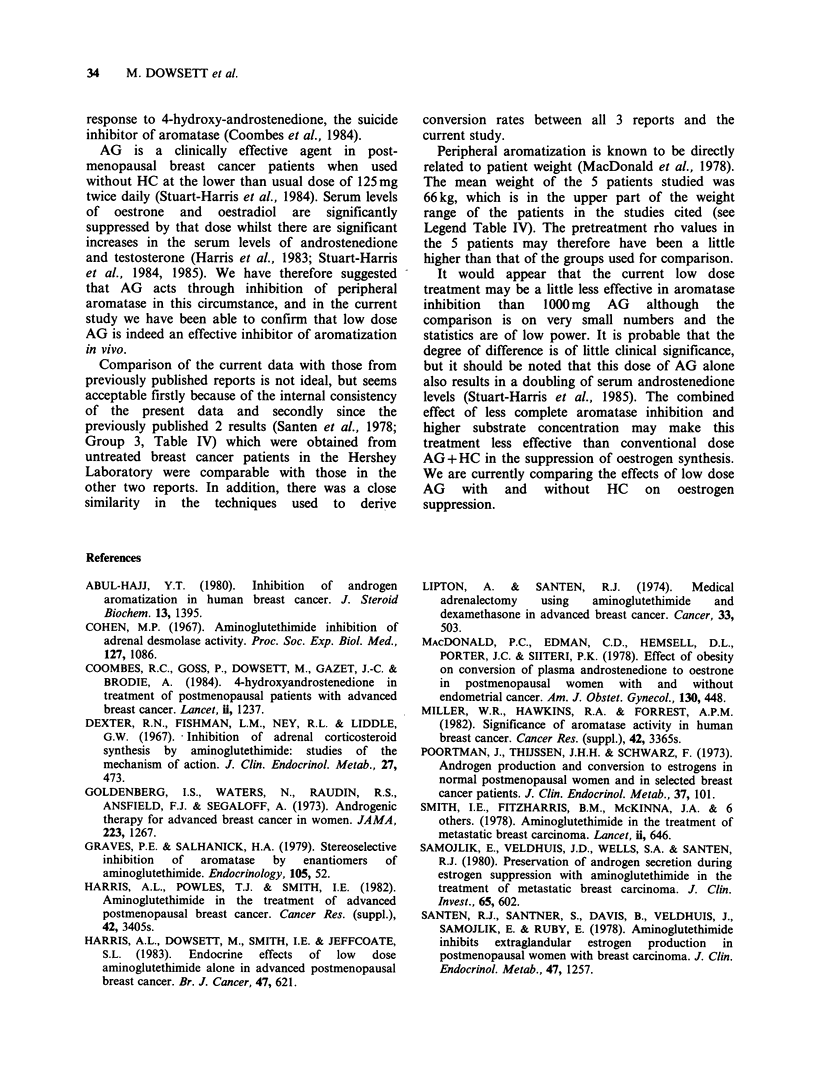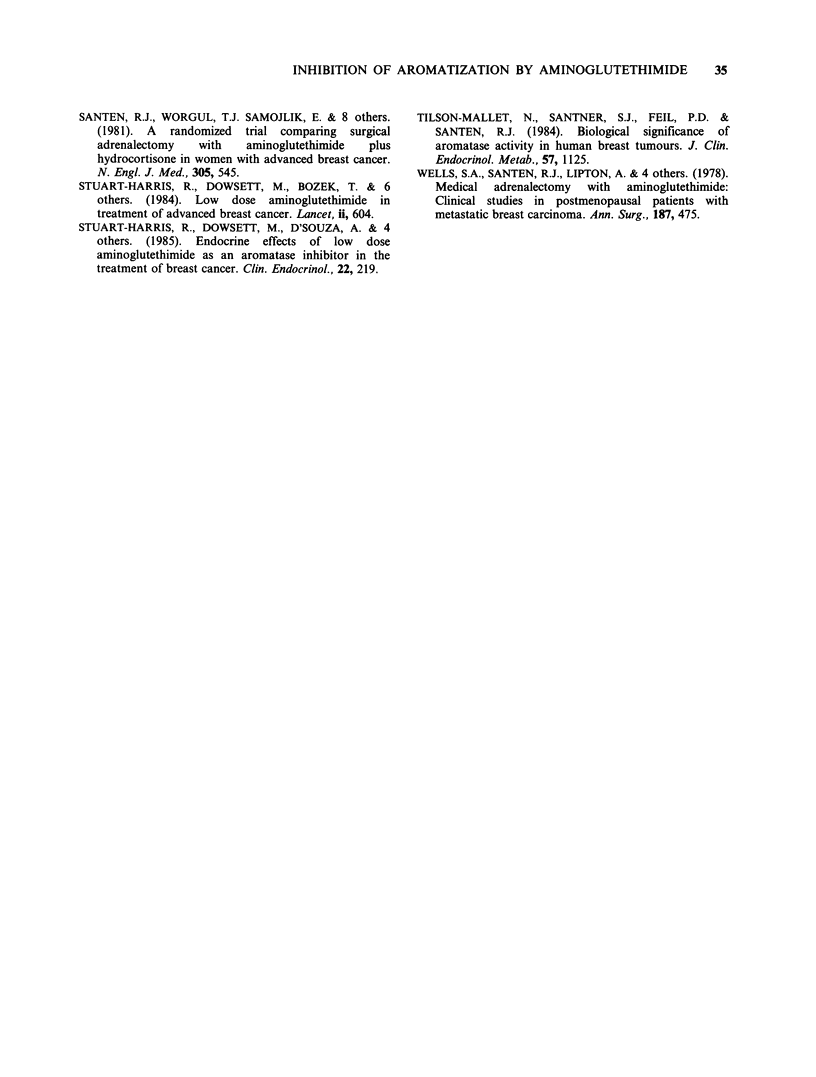# Effective inhibition by low dose aminoglutethimide of peripheral aromatization in postmenopausal breast cancer patients.

**DOI:** 10.1038/bjc.1985.144

**Published:** 1985-07

**Authors:** M. Dowsett, S. J. Santner, R. J. Santen, S. L. Jeffcoate, I. E. Smith

## Abstract

Aminoglutethimide without glucocorticoid has been shown to be a clinically effective treatment for postmenopausal breast cancer in low dosage (250 mg day-1). The mechanism of action of this approach is thought to be the inhibition of peripheral aromatase, the enzyme which converts androstenedione to oestrone. The activity of this enzyme was measured in vivo by injection with 3H-androstenedione and 14C-oestrone and found to be 0.20% +/- 0.05 in 5 patients on low dose AG therapy. In comparison with previously published data this demonstrates a 92% inhibition of peripheral aromatase activity confirming aromatase inhibition as a viable aim in the endocrine treatment of breast cancer.


Br. J. Cancer (1985), 52, 31–35

### Effective inhibition by low dose aminoglutethimide of peripheral aromatization in postmenopausal breast cancer patients

M. Dowsett[1], S.J. Santner[2], R.J. Santen[2], S.L. Jeffcoate[1] & I.E. Smith[3]

[1]*Department of Biochemical Endocrinology, Chelsea Hospital for Women, Dovehouse Street, London SW3 6LT, UK;* [2]*Department of Medicine, Division of Endocrinology, Milton S. Hershey Medical Center, Pennsylvania State University College of Medicine, Hershey, Pennsylvania 17033, USA and* [3]*Medical Breast Unit, Royal Marsden Hospital, Fulham Road, London SW3 6JJ, UK.*

Aminoglutethimide without glucocorticoid has been shown to be a clinically effective treatment for postmenopausal breast cancer in low dosage (250 mg day$^{-1}$). The mechanism of action of this approach is thought to be the inhibition of peripheral aromatase, the enzyme which converts androstenedione to oestrone. The activity of this enzyme was measured *in vivo* by injection with $^{3}$H-androstenedione and $^{14}$C-oestrone and found to be 0.20% ± 0.05 in 5 patients on low dose AG therapy. In comparison with previously published data this demonstrates a 92% inhibition of peripheral aromatase activity confirming aromatase inhibition as a viable aim in the endocrine treatment of breast cancer.

Aminoglutethimide (AG) is a clinically effective endocrine treatment for advanced postmenopausal breast cancer (Lipton *et al.*, 1974; Smith *et al.*, 1978; Santen *et al.*, 1981; Harris *et al.*, 1982), in which it has been used almost exclusively in doses of 750–1000 mg day$^{-1}$, in combination with hydrocortisone (HC). This therapeutic regime was derived with the aim of suppressing adrenal androgen synthesis (Lipton *et al.*, 1974; Wells *et al.*, 1978) which was expected to result from previously reported inhibition by AG of the conversion of cholesterol to pregnenolone by 20,22-desmolase (Cohen, 1967; Dexter *et al.*, 1967). HC was added to the regime to prevent a rise in adrenocorticotrophic hormone, which would result from suppression of cortisol synthesis, and which might overcome the enzyme block (Wells *et al.*, 1978).

More recently it has become apparent that AG + HC has little effect on serum levels of adrenal androgens (Samojlik *et al.*, 1980; Harris *et al.*, 1983) and its clinical effectiveness in breast cancer is probably due to inhibition by AG of peripheral (Santen *et al.*, 1978) and perhaps intratumoural aromatization of androgens to oestrogens (Abul-Hajj, 1980; Miller *et al.*, 1982; Tilson-Mallet *et al.*, 1984). The inhibitory potency of AG on aromatase *in vitro* has been shown to be at least ten-fold greater than on the 20,22 desmolase (Graves & Salhanick, 1979) and this has led to the examination of the clinical and endocrine effectiveness of AG at low dosage (250 mg day$^{-1}$) without HC (Harris *et al.*, 1983; Stuart-Harris *et al.*, 1984, 1985). When used in a dose of 1000 mg (+40 mg HC) day$^{-1}$, AG was found to inhibit peripheral aromatase activity *in vivo* by at least 95% (Santen *et al.* 1978). We report here the measurement of this activity *in vivo* in postmenopausal breast cancer patients undergoing treatment at a lower dosage (250 mg day$^{-1}$).

Correspondence: M. Dowsett.
Received 18 February 1985; and in revised form 18 March 1985.

### Patients and methods

### *Radioactive injections*

Five patients treated with AG 125 mg twice daily were studied. These patients were part of a clinical study of the effectiveness of this treatment (Stuart-Harris *et al.*, 1984) and their clinical details are given in Table I. Approval from the local ethical committee, informed consent and a DHSS licence were obtained before commencement of the study. Each patient received by bolus intravenous injection 120 $\mu$Ci of [7$\alpha$-$^{3}$H] androstenedione ($\Delta^4$A, 30 Ci mmol$^{-1}$, New England Nuclear) and 1 $\mu$Ci of [4-$^{14}$C] oestrone ($E_1$, 55 mCi mmol$^{-1}$, Amersham International) in 58 ml isotonic saline between 10.30 and 12.00. One ml of this mixture was retained for estimation of $^{3}$H:$^{14}$C ratio. All urine passed during the next 72 h was collected and was kept at −20°C until analysis.

© The Macmillan Press Ltd., 1985

**Table I** Patient details at time of study

| Patient | *Age (years)* | *Weight (kg)* | *Time on AG treatment (months)* | *Time since primary diagnosis (years)* | *Previous chemotherapy* | *Sites of disease* | *Response* |
|---|---|---|---|---|---|---|---|
| 1 | 58 | 78 | 14 | 8 | — | Chest wall | Stable |
| 2 | 70 | 70 | 14 | 9 | — | Pleura | Partial |
| 3 | 69 | 56 | 19 | 24 | — | Pleura | Partial |
| 4 | 76 | 54 | 17 | 4 | (i) Tamoxifen (ii) Cyclophosphamide | Skin/lymph nodes | Complete |
| 5 | 70 | 73 | 11 | 1 | — | Skin, bone | Partial |

Response assessed according to standard UICC criteria.

*Purification of urinary oestrone*

The initial stages of purification (i.e. Amberlite chromatography, $\beta$-glucuronidase digestion, ethyl acetate extraction and phenolic extraction) were as previously described (Santen *et al.,* 1978). After nearly drying the residues from the phenolic extract, they were subjected to thin layer chromatography (TLC) as outlined in Table II. The first system listed was used twice in 4 of the 5 samples; its function was to remove enough extraneous material so that the samples would not smear in subsequent TLCs. The final system was preceded by an acetylation, performed by adding 12 drops of pyridine and 6 drops of acetic anhydride to the sample and incubating overnight at room temperature.

**Table II** Solvent systems used in the thin-layer chromatography steps in the purification of oestrone

| *System no.* | *System composition* |
|---|---|
| I | Benzene:Ethanol (80:20) |
| II | Methylene chloride:Ether (90:10) |
| III | Methylene chloride:Methanol (95:5) |
| IV | Chloroform:Ethyl acetate (80:20) |
| V | Methylene chloride:Ether (96:4) |

*Calculation of $\Delta^4A$ to $E_1$ rho values*

$^3H$:$^{14}C$ ratio of $E_1$ was determined after each chromatographic purification step. $^{14}C$ c.p.m. were corrected for background and $^3H$ c.p.m. for spill-over from $^{14}C$ which was 14.7% in these studies. The conversion of $\Delta^4A$ to $E_1$ ($\rho$) was calculated according to the formula $\%(\rho) = {}^3H:{}^{14}C$ ratio urinary $E_1 \div {}^3H:{}^{14}C$ ratio injection mixture $\times 100$.

The $^3H$:$^{14}C$ ratio after the final purification step was used in each case.

### Results

The $^3H$:$^{14}C$ ratio of the injection mixture and of urinary $E_1$ at each stage of purification are shown in Table III together with the calculated $\rho$ values. The $^3H$:$^{14}C$ ratio of urinary $E_1$ was essentially constant between the last 2 chromatographic steps. The mean value for the conversion of $\Delta^4A$ to $E_1$ was $0.20 \pm 0.56\%$ (s.d.).

It was not possible to determine pretreatment values in these patients but for comparative purposes values may be drawn from previously published studies (Poortman *et al.,* 1973; MacDonald *et al.,* 1978; Santen *et al.,* 1978) which used the same methodology. These data are shown in Table IV, and are plotted in Figure 1 together with the $\rho$ values from patients in the current study, and 2 patients treated with 1000 mg AG from a previous study (Santen *et al.,* 1978). A mean $\rho$ value of $2.5\% \pm 0.8$ (s.d.) may be derived from the pooled data. Comparison (unpaired $t$-test) of these values with those obtained in the 5 patients treated with low dose AG shows that the latter group of values were significantly lower ($P<0.001$, $t=6.77$) showing a mean 92% inhibition of conversion of $\Delta^4A$ to $E_1$. Comparison of the values in the 5 treated patients against the 2 obtained previously in patients treated with 1000 mg AG daily ($\rho=0.01$, 0.08) showed these 2 values to be significantly lower ($P<0.05$, $t=2.64$).

### Discussion

Aromatase inhibition would appear to be an effective mechanism for the endocrine treatment of postmenopausal breast cancer. This has been previously suggested by demonstration of clinical responses to testololactone (Goldenberg *et al.,* 1973) and aminoglutethimide (Santen *et al.,* 1981) and recently confirmed by the demonstration of

**Table III** Determination of % rho-values for conversion of $\Delta^4A$ to $E_1$. $^3H:^{14}C$ ratios were determined after each chromatographic step and the % rho value was derived as $^3H:^{14}C$ ratio after the final chromatographic step × 100/$^3H:^{14}C$ ratio of the injection mixture

| *Subject no.* | *1* | | | *2* | | | *3* | | | *4* | | | *5* | | |
|---|---|---|---|---|---|---|---|---|---|---|---|---|---|---|---|
| | *c.p.m.* | | | *c.p.m.* | | | *c.p.m.* | | | *c.p.m.* | | | *c.p.m.* | | |
| *Step* | $^3H$ | $^{14}C$ | $^3H:^{14}C$ | $^3H$ | $^{14}C$ | $^3H:^{14}C$ | $^3H$ | $^{14}C$ | $^3H:^{14}C$ | $^3H$ | $^{14}C$ | $^3H:^{14}C$ | $^3H$ | $^{14}C$ | $^3H:^{14}C$ |
| Phenolic extract | 9763 | 1183 | 8.25 | 4152 | 752 | 5.51 | 6843 | 1147 | 5.96 | 3992 | 1097 | 3.63 | 16922 | 844 | 20.04 |
| TLC—IA | 1638 | 128 | 12.81 | 138 | 34.4 | 4.00 | 407 | 61.4 | 6.62 | 1342 | 341 | 3.93 | 813 | 27.2 | 29.87 |
| TLC—IB | 945 | 115 | 8.24 | 103 | 33.5 | 3.06 | — | — | — | 414 | 92.7 | 4.46 | 1472 | 46.4 | 31.73 |
| TLC—II | 423 | 491 | 0.86 | 71.7 | 120 | 0.59 | 242 | 235 | 1.02 | 158 | 357 | 0.44 | 66.0 | 106 | 0.62 |
| TLC—III | 1236 | 1532 | 0.80 | 21.9 | 290 | 0.07 | 107 | 455 | 0.23 | 102 | 433 | 0.23 | 103 | 343 | 0.29 |
| TLC—IV | 144 | 1145 | 0.12 | 10.5 | 189 | 0.05 | 43.0 | 314 | 0.13 | 31.0 | 245 | 0.12 | 40.2 | 230 | 0.17 |
| TLC—V | 71.3 | 545 | 0.13 | 22.3 | 193 | 0.12 | 54.0 | 347 | 0.15 | 36.5 | 279 | 0.13 | 50.7 | 261 | 0.19 |
| $^3H:^{14}C$ injection mixture | 66.3 | | | 99.0 | | | 57.6 | | | 69.0 | | | 95.4 | | |
| % rho-value | 0.20 | | | 0.12 | | | 0.27 | | | 0.19 | | | 0.20 | | |

**Table IV** % conversion $\Delta^4A$ to $E_1$ measured in the urine of untreated postmenopausal women.

| *Patient* | *% conversion $\Delta^4A$ to $E_1$* | *Patient* | *% conversion $\Delta^4A$ to $E_1$* |
|---|---|---|---|
| *Group 1*[a] | | *Group 2*[b] | |
| 1 | 2.1 | 1 | 1.6 |
| 2 | 1.3 | 2 | 1.5 |
| 3 | 3.8 | 3 | 2.4 |
| 4 | 2.8 | 4 | 3.3 |
| 5 | 3.3 | 5[d] | 2.4 |
| 6 | 1.9 | 6 | 2.4 |
| 7 | 3.0 | 7 | 2.7 |
| 8 | 1.6 | 8 | 3.8 |
| 9[d] | 2.8 | 9 | 2.1 |
| 10[d] | 3.7 | | |
| 11[d] | 2.4 | *Group 3*[c] | |
| 12[d] | 3.0 | 1[d] | 1.9 |
| 13[d] | 2.9 | 2[d] | 1.1 |
| 14[d] | 2.8 | | |
| 15[d] | 2.6 | | |

[a]Poortman *et al.* (1973), weight range 41–71 kg;
[b]MacDonald *et al.* (1978), excluding patients with ovarian serous cystadenocarcinomas, weight range 46–73 kg;
[c]Santen *et al.* (1978), weight range 45–63 kg;
[d]Breast cancer patients.

**Figure 1** % conversion of $\Delta^4A$ to $E_1$ in postmenopausal females. Left-hand column—no treatment: (●) normals, (○) breast cancer patients, (Ⓐ) breast cancer patients before 1000 mg AG day$^{-1}$.
Right-hand column—breast cancer patients on AG: (Ⓐ) 1000 mg day$^{-1}$, (Ⓛ) 250 mg day$^{-1}$.

response to 4-hydroxy-androstenedione, the suicide inhibitor of aromatase (Coombes et al., 1984).

AG is a clinically effective agent in post-menopausal breast cancer patients when used without HC at the lower than usual dose of 125 mg twice daily (Stuart-Harris et al., 1984). Serum levels of oestrone and oestradiol are significantly suppressed by that dose whilst there are significant increases in the serum levels of androstenedione and testosterone (Harris et al., 1983; Stuart-Harris et al., 1984, 1985). We have therefore suggested that AG acts through inhibition of peripheral aromatase in this circumstance, and in the current study we have been able to confirm that low dose AG is indeed an effective inhibitor of aromatization *in vivo*.

Comparison of the current data with those from previously published reports is not ideal, but seems acceptable firstly because of the internal consistency of the present data and secondly since the previously published 2 results (Santen et al., 1978; Group 3, Table IV) which were obtained from untreated breast cancer patients in the Hershey Laboratory were comparable with those in the other two reports. In addition, there was a close similarity in the techniques used to derive conversion rates between all 3 reports and the current study.

Peripheral aromatization is known to be directly related to patient weight (MacDonald et al., 1978). The mean weight of the 5 patients studied was 66 kg, which is in the upper part of the weight range of the patients in the studies cited (see Legend Table IV). The pretreatment rho values in the 5 patients may therefore have been a little higher than that of the groups used for comparison.

It would appear that the current low dose treatment may be a little less effective in aromatase inhibition than 1000 mg AG although the comparison is on very small numbers and the statistics are of low power. It is probable that the degree of difference is of little clinical significance, but it should be noted that this dose of AG alone also results in a doubling of serum androstenedione levels (Stuart-Harris et al., 1985). The combined effect of less complete aromatase inhibition and higher substrate concentration may make this treatment less effective than conventional dose AG+HC in the suppression of oestrogen synthesis. We are currently comparing the effects of low dose AG with and without HC on oestrogen suppression.

### References

ABUL-HAJJ, Y.T. (1980). Inhibition of androgen aromatization in human breast cancer. *J. Steroid Biochem.* **13**, 1395.

COHEN, M.P. (1967). Aminoglutethimide inhibition of adrenal desmolase activity. *Proc. Soc. Exp. Biol. Med.*, **127**, 1086.

COOMBES, R.C., GOSS, P., DOWSETT, M., GAZET, J.-C. & BRODIE, A. (1984). 4-hydroxyandrostenedione in treatment of postmenopausal patients with advanced breast cancer. *Lancet*, **ii**, 1237.

DEXTER, R.N., FISHMAN, L.M., NEY, R.L. & LIDDLE, G.W. (1967). Inhibition of adrenal corticosteroid synthesis by aminoglutethimide: studies of the mechanism of action. *J. Clin. Endocrinol. Metab.*, **27**, 473.

GOLDENBERG, I.S., WATERS, N., RAUDIN, R.S., ANSFIELD, F.J. & SEGALOFF, A. (1973). Androgenic therapy for advanced breast cancer in women. *JAMA*, **223**, 1267.

GRAVES, P.E. & SALHANICK, H.A. (1979). Stereoselective inhibition of aromatase by enantiomers of aminoglutethimide. *Endocrinology*, **105**, 52.

HARRIS, A.L., POWLES, T.J. & SMITH, I.E. (1982). Aminoglutethimide in the treatment of advanced postmenopausal breast cancer. *Cancer Res.* (suppl.), **42**, 3405s.

HARRIS, A.L., DOWSETT, M., SMITH, I.E. & JEFFCOATE, S.L. (1983). Endocrine effects of low dose aminoglutethimide alone in advanced postmenopausal breast cancer. *Br. J. Cancer*, **47**, 621.

LIPTON, A. & SANTEN, R.J. (1974). Medical adrenalectomy using aminoglutethimide and dexamethasone in advanced breast cancer. *Cancer*, **33**, 503.

MACDONALD, P.C., EDMAN, C.D., HEMSELL, D.L., PORTER, J.C. & SIITERI, P.K. (1978). Effect of obesity on conversion of plasma androstenedione to oestrone in postmenopausal women with and without endometrial cancer. *Am. J. Obstet. Gynecol.*, **130**, 448.

MILLER, W.R., HAWKINS, R.A. & FORREST, A.P.M. (1982). Significance of aromatase activity in human breast cancer. *Cancer Res.* (suppl.), **42**, 3365s.

POORTMAN, J., THIJSSEN, J.H.H. & SCHWARZ, F. (1973). Androgen production and conversion to estrogens in normal postmenopausal women and in selected breast cancer patients. *J. Clin. Endocrinol. Metab.*, **37**, 101.

SMITH, I.E., FITZHARRIS, B.M., MCKINNA, J.A. & 6 others. (1978). Aminoglutethimide in the treatment of metastatic breast carcinoma. *Lancet*, **ii**, 646.

SAMOJLIK, E., VELDHUIS, J.D., WELLS, S.A. & SANTEN, R.J. (1980). Preservation of androgen secretion during estrogen suppression with aminoglutethimide in the treatment of metastatic breast carcinoma. *J. Clin. Invest.*, **65**, 602.

SANTEN, R.J., SANTNER, S., DAVIS, B., VELDHUIS, J., SAMOJLIK, E. & RUBY, E. (1978). Aminoglutethimide inhibits extraglandular estrogen production in postmenopausal women with breast carcinoma. *J. Clin. Endocrinol. Metab.*, **47**, 1257.

SANTEN, R.J., WORGUL, T.J. SAMOJLIK, E. & 8 others. (1981). A randomized trial comparing surgical adrenalectomy with aminoglutethimide plus hydrocortisone in women with advanced breast cancer. *N. Engl. J. Med.*, **305**, 545.

STUART-HARRIS, R., DOWSETT, M., BOZEK, T. & 6 others. (1984). Low dose aminoglutethimide in treatment of advanced breast cancer. *Lancet*, **ii**, 604.

STUART-HARRIS, R., DOWSETT, M., D'SOUZA, A. & 4 others. (1985). Endocrine effects of low dose aminoglutethimide as an aromatase inhibitor in the treatment of breast cancer. *Clin. Endocrinol.*, **22**, 219.

TILSON-MALLET, N., SANTNER, S.J., FEIL, P.D. & SANTEN, R.J. (1984). Biological significance of aromatase activity in human breast tumours. *J. Clin. Endocrinol. Metab.*, **57**, 1125.

WELLS, S.A., SANTEN, R.J., LIPTON, A. & 4 others. (1978). Medical adrenalectomy with aminoglutethimide: Clinical studies in postmenopausal patients with metastatic breast carcinoma. *Ann. Surg.*, **187**, 475.